# University Law Suit

**Published:** 1854-07

**Authors:** 


### UNIVERSITY LAW-SUIT.

Most of our readers are aware that a suit has been for some time pending in the Court of Appeals in Kentucky, involving the right of management in the property and affairs of the University of Louisville. It was a suit of great magnitude. The interests involved were no less than the stability and permanent success of the institution. The question was believed to be a vital one, at least to the medical department. It had attracted very wide attention, and much solicitude has been felt about its decision. We are happy to be able to announce, that the Court of Appeals have affirmed the decision of the Court below, and that, consequently, the property and administration of the school remain in the hands of the old Board of Trustees.

A brief narrative of the origin and progress of this suit will probably not be uninteresting to our readers. To be understood, it will be necessary to go back to the foundation of the Louisville Medical Institute.

This School was chartered by the Legislature of Kentucky in 1833, but was not fully organized until 1837. In the spring of that year, at a meeting of the citizens of Louisville, it was resolved, that there ought to be a College in the city, with both medical and law departments, and that the medical department ought to be put in immediate operation. The City Council was requested by this meeting to grant the square bounded by Eighth and Ninth, and Chestnut and Magazine streets, to the Board of Managers of the Medical Institute, and to erect thereon necessary buildings for a medical school, and purchase a library and apparatus, at the expense of the city of Louisville. According to these resolutions the square was granted to the Managers of the Medical Institute, and an appropriation of $20,000 was made for the purchase of books, apparatus, and other materials of medical instruction. During the summer following the College edifice was erected at a cost somewhat exceeding $50,000.

The Board of Managers of the Medical Institute, according to their charter, continued in office for life, and filled vacancies as they occurred in the Board—a *close corporation*, as it is termed. This Board continued to have control of the institution until 1846, when, according to the terms of the grant of the City Council, the property and government passed into the hands of the Trustees of the University, just chartered, of which it became the medical department. The Board of Trustees was no longer a perpetual, self-appointing body, but was elected by the City Council, for ten years, two members going out every two years. This concession was made to the prejudice against close corporations, and while by the change it was meant to bring the institution more under the control of the city, the tenure of office was thought to be long enough to prevent any sudden or violent change in the character of the Board of Trustees. In the charter of the University it was also wisely provided, that the fees of every department should be applied to its own use.

A Law department was organized by the Board of Trustees, in 1846, and has been eminently successful. In 1850 a Convention was called by the citizens of Louisville to frame a new city charter, and the convention undertook to make various important modifications in the charter of the University. The new charter was adopted by the people, and afterwards, in 1851, enacted by the Legislature. By this charter, the trustees are elected directly by the people, and possess unlimited control over the fees and revenues of all the departments of the University. Many of the ablest jurists in the Legislature believing that the new charter was unconstitutional and unjust, refused to vote for it except with a proviso, that it should be submitted for adjudication to the Jefferson Circuit Court. Judge Goodloe heard the case in 1852, which was ably argued by Judge Bodley, and Gwin Page, Esq., for the city, and by Judge Nicholas, and Bland Ballard, Esq., for the University, and decided the clause relating to the University to be unconstitutional. The city appealed, and the Court of Appeals, after hearing the same learned counsel in the case, and having it for more than two years under consideration, have just confirmed the decision of the Jefferson Court. This decision is final. The tribunal which has now decided the case is the court of last resort, and the controversy is therefore at an end.

No doubt, it is well for the University that the case has had a judicial decision, which settles the question forever. It had been started again and again, first in one shape and then in another, to the great annoyance and detriment of the medical school; and until the Courts had spoken it is doubtful whether the school would ever have had rest from those who were constantly seeking to deprive it of its endowment and change its government and organization. The plea which they continually put forth was, that the medical school was too profitable to its professors, and that under a different arrangement this department might be made to sustain the literary department of the University. Many persons were captivated by this scheme, but all who were informed on the subject saw that it was a project, not to establish an academical department, but, in reality, to break down the medical department.

No one can fail to see that this would have been the result of the enforcement of the new charter. A medical school must have permanence, stability. There must be a fair prospect that its teachers, if acceptable and competent, will be the same from year to year, for a reasonable period of time. Its faculty must have a feeling of security, a freehold in their offices, not to be disturbed except for sufficient cause. They must also have the promise of liberal remuneration for their services. No school is likely to prosper unless its professors receive from it an adequate support. But under the condemned charter none of this security, either of office or emolument, could have existed. The Trustees, one half to be elected every year, would have come into office with prejudices or prepossessions if not pledges, regarding the medical school. Taxation would have been the first thing, and as the authority of the Board in this respect was to be unrestricted, it could not be foreseen how heavy the burden would have been made. Any amount of the profits of the professors that seemed to the Board proper, might have been diverted to the use of other departments. This would have been an intolerable grievance; but it was not the worst contemplated by the framers of the new charter. One half of the Board of Trustees being eligible by the people annually, the school would have been kept in a state of unceasing turmoil. The question would have been continually started by those interested in a change, "are the professors in the school the best that could be found?" There would thus have arisen a perpetual contest about the professorships, which could not have been otherwise than disastrous to the institution.

It was with such views of the tendency of the new charter as these, that the friends of the University opposed its adoption. They regarded it as a scheme artfully devised for the overthrow of the medical department. But it has failed, and litigation respecting the government and property of the institution is now at an end. The question cannot be revived; it has gone through all the processes known to our jurisprudence, and the decision now rendered in the case is final.

Everything connected with new countries is said to be peculiarly subject to fluctuation and change. Medical schools, we are very sure, can claim no exemption from the general law. Instability has been the striking characteristic of most of those which have risen west of the Alleghanies. We are not about to write their histories. A happier day, we trust, awaits these institutions, and is already beginning to dawn upon them. The period of mutability, we may hope, is passing away, and an era of stability and progress is about to be inaugurated. The medical school at Louisville was established under circumstances favorable to perpetuity and enduring success. The citizens decreed its foundation, and voted it a munificent endowment. It was provided with a spacious edifice, a good library, and ample apparatus; it was unencumbered by debt, and had an income for the regular increase of its facilities for teaching. Its growth was rapid. In a few years its class grew to be one of the largest in the country; but it was not long before it was threatened with danger. The third summer after the school went into operation, a project was set on foot to change its form of government, and transfer it to a new Board of Trustees. For fourteen years it has had to meet these assaults, made first in one shape and then in another, but it has successfully resisted all the attempts at revolution, and has, at last, a fair prospect of repose. Relieved from the incubus of a lawsuit which threatened its very existence, it has now nothing to do but go on in the quiet performance of its legitimate functions, increasing and improving its means and methods of instruction, keeping steadily in view, as we trust it always will, the advancement of medical science, and the dignity and honor of the medical profession.

